# Midget retinal ganglion cell dendritic and mitochondrial degeneration is an early feature of human glaucoma

**DOI:** 10.1093/braincomms/fcz035

**Published:** 2019-11-28

**Authors:** James R Tribble, Asta Vasalauskaite, Tony Redmond, Robert D Young, Shoaib Hassan, Michael P Fautsch, Frank Sengpiel, Pete A Williams, James E Morgan

**Affiliations:** 1 School of Optometry and Vision Sciences, Cardiff University, Cardiff, CF24 4HQ Wales, UK; 2 Department of Clinical Neuroscience, Division of Eye and Vision, St. Erik Eye Hospital, Karolinska Institutet, 112 82 Stockholm, Sweden; 3 School of Biosciences, Cardiff University, Cardiff, CF10 3AX Wales, UK; 4 School of Medicine, Cardiff University, Heath Park, Cardiff, CF14 4XW Wales, UK; 5 Department of Ophthalmology, Mayo Clinic, Rochester, Minnesota, USA

**Keywords:** glaucoma, retinal ganglion cell, electron microscopy, dendrite, mitochondria

## Abstract

Glaucoma is characterized by the progressive dysfunction and loss of retinal ganglion cells. However, the earliest degenerative events that occur in human glaucoma are relatively unknown. Work in animal models has demonstrated that retinal ganglion cell dendrites remodel and atrophy prior to the loss of the cell soma. Whether this occurs in human glaucoma has yet to be elucidated. Serial block face scanning electron microscopy is well established as a method to determine neuronal connectivity at high resolution but so far has only been performed in normal retina from animal models. To assess the structure–function relationship of early human glaucomatous neurodegeneration, regions of inner retina assessed to have none-to-moderate loss of retinal ganglion cell number were processed using serial block face scanning electron microscopy (*n *=* *4 normal retinas, *n *= 4 glaucoma retinas). This allowed detailed 3D reconstruction of retinal ganglion cells and their intracellular components at a nanometre scale. In our datasets, retinal ganglion cell dendrites degenerate early in human glaucoma, with remodelling and redistribution of the mitochondria. We assessed the relationship between visual sensitivity and retinal ganglion cell density and discovered that this only partially conformed to predicted models of structure–function relationships, which may be affected by these early neurodegenerative changes. In this study, human glaucomatous retinal ganglion cells demonstrate compartmentalized degenerative changes as observed in animal models. Importantly, in these models, many of these changes have been demonstrated to be reversible, increasing the likelihood of translation to viable therapies for human glaucoma.

## Teaser

We demonstrate midget retinal ganglion cell dendritic degeneration coupled with remodelling and redistribution of mitochondria as features of human glaucoma. These changes may contribute to changes in visual sensitivity during disease progression and extend the similarities between human glaucoma patients and animal models of glaucoma.

## Graphical Abstract

**Graphical Abstract:**
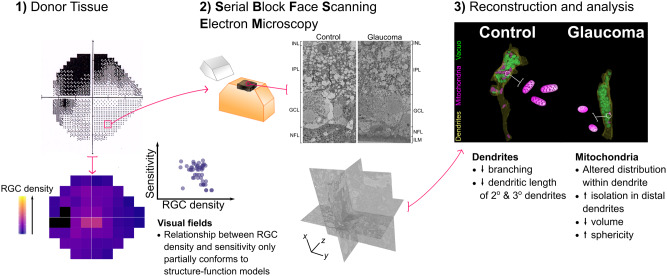

## Introduction

With an estimated 70 million patients worldwide, glaucoma remains a leading cause of irreversible blindness and a major economic burden (Tham *et al.*, 2014). The earliest detectable neurodegenerative changes in human patients are yet to be fully elucidated, but their discovery could provide novel biomarkers to support early diagnosis and treatment.

Glaucoma is characterized by the progressive dysfunction and death of retinal ganglion cells. Age, genetics, and elevated intraocular pressure are prominent risk factors for the development of human glaucoma. Animal models of glaucomatous ocular hypertension (which recapitulate the elevated intraocular pressure risk factor seen in many glaucoma patients) have demonstrated that retinal ganglion cell mitochondrial abnormalities (Williams *et al.*, 2017*b*), synapse loss (Della Santina *et al.*, 2013; Berry *et al.*, 2015; Williams *et al.*, 2016), and dendritic atrophy (Williams *et al.*, 2013*a*) precede cell death. These disease features have been demonstrated across model species; in mouse (Leung *et al.*, 2011; Feng *et al.*, 2013; Williams *et al.*, 2013*a*; Berry *et al.*, 2015), rat (Morgan *et al.*, 2006; Urcola *et al.*, 2006; Williams *et al.*, 2016), cat (Shou *et al.*, 2003), and non-human primate (Weber *et al.*, 1998; Morgan *et al.*, 2000). Importantly, whereas axon regeneration does not occur in the mammalian optic nerve, retinal ganglion cell dendritic and synaptic plasticity, regrowth, and re-innervation could underpin visual recovery during early human glaucoma. However, there is no evidence that these degenerative changes occur as an early feature of human glaucoma, with only a single study showing dendritic loss in eyes, which had progressed to complete blindness (Pavlidis *et al.*, 2003). Current evidence of retinal ganglion cell death in glaucoma comes from cell counts in donor tissue (Quigley *et al.*, 1989), live imaging of apoptosis (Cordeiro *et al.*, 2017), and retinal nerve fibre layer thinning (retinal ganglion cell axons in the inner retina) measured by optical coherence tomography (OCT; Raza *et al.*, 2011). Clinical measures of vision loss come from functional tests, in which deficits at the level of single retinal ganglion cells could be masked through the summation of outputs in the visual centres of the brain (Redmond *et al.*, 2010; Mulholland *et al.*, 2015).

In routine clinical practice, visual function is measured in glaucoma by Standard Automated Perimetry in which spot stimuli of modulated luminance are presented to determine visual sensitivity at specific locations in the visual field. Although this technique is regarded as a gold standard clinical test of visual function in glaucoma, it has poor sensitivity to early disease (Tafreshi *et al.*, 2009) and high variability confounding the identification of statistically significant visual deterioration (Artes *et al.*, 2002). The ‘hockey stick’ model of Swanson *et al.* (2004) describes the relationship between visual field sensitivity and retinal ganglion cell density in healthy eyes. It predicts that the rate of change in sensitivity with respect to retinal ganglion cell number is low when the stimulus is larger than the critical summation area (2.5 dB loss per log unit reduction in cell number) and increases when the stimulus is smaller than the critical summation area (10 dB loss per log unit reduction in cell number; i.e. a 1:1 relationship since 1 dB = 0.1 log unit attenuation of stimulus luminance from the maximum). Since the perimetric stimuli are of fixed area and the critical summation area is known to enlarge in early glaucoma (Redmond *et al.*, 2010), the ‘hockey stick’ model predicts an initial slow decline in visual field sensitivity when the remaining retinal ganglion cell density is high, followed by a steep decline once substantial retinal ganglion cell loss has occurred. When sensitivity loss is mild, early neurodegenerative changes may be masked by spatial summation and within- and between-test variabilities when assessed by Standard Automated Perimetry.

To assess the structure–function relationship of early human glaucomatous neurodegeneration, we used two-photon imaging of regions of whole human control and glaucomatous retina to determine the relationship between retinal ganglion cell loss and visual field sensitivity (as assessed by Standard Automated Perimetry). Regions of inner retina assessed to have none-to-moderate cell loss and visual deficit were processed using serial block face scanning electron microscopy (SBFSEM) to generate detailed 3D reconstructions of retinal ganglion cells and their intracellular components at a nanometre scale. This automated approach has been used to great effect to generate and analyse large-scale retinal connectomes in normal animal tissue (Briggman *et al.*, 2011; Helmstaedter *et al.*, 2013) and to investigate pathophysiological changes to single neurons in other neurodegenerations (Yamasaki *et al.*, 2014; Giacci *et al.*, 2018). We applied these methods to quantify mitochondrial and dendritic abnormalities in regions of no-to-moderate visual deficit in the human glaucomatous retina. To date, no studies of human glaucomatous retinal ganglion cells have been undertaken using SBFSEM, and as such, this study represents the first attempt to resolve human retinal ganglion cells and their intracellular components in 3D at this scale.

## Materials and methods

### Human tissue

Donor tissue was obtained from the Minnesota Lions Eye Bank (St. Paul, MN, USA) in accordance with local ethical approval. Donor eyes (*n *=* *4 eyes; mean age 74.5 years) with primary open angle glaucoma and controls (*n *=* *8 eyes; mean age 81.6 years) were used. Posterior globes were fixed in 4% paraformaldehyde in 0.1 M phosphate buffer within 24 h of the recorded time of death and then transferred to Cardiff University, UK, and stored at 4°C until used. Work carried out in Cardiff was in compliance with the UK Human Tissue Act 2004. Donor information is summarized in Table 1. The four glaucomatous eyes had corresponding visual field tests (Humphrey Field Analyzer; HFA II, Carl Zeiss Meditec, Dublin, CA, USA; SITA-Standard, 24-2 test pattern, Goldmann III stimulus, and 200 ms duration) undertaken 7–21 months before death.

**Table 1:** Donor details

Sample ID	Eye	Condition	Sex	Age	Time from death to fixation (h)	Time in fixation prior to study (years)	Time from visual field test to death (months)	IOP (mmHg) at (a) 12, (b) 6 and (c) 0 months prior to field test	Usage: (1) cell counts and (2) SBFSEM
GL239	L	Glaucoma (POAG)	M	77	3.83	4–3.5	21	(a) 10(b) 13(c) 17	1, 2
GL239	R	Glaucoma (POAG)	M	77	3.83	4–3.5	21	(a) 13(b) 14(c) 17	1, 2
GL277	L	Glaucoma (POAG)	F	72	2.3	1.5–3.5	7	(a) NA(b) 21(c) 13	1, 2
GL277	R	Glaucoma (POAG)	F	72	2.3	1.5–3.5	7	(a) NA(b) 21(c) 13	1, 2
14-0625	R	Control	F	83	6.5	1.5–3.5	NA	NA	1, 2
14-1137	L	Control	F	83	9	1.5–3.5	NA	NA	2
14-0899	L	Control	M	86	6.5	1.5–3.5	NA	NA	1, 2
16-1025	R	Control	M	83	7.33	2	NA	NA	2
14-1357	L	Control	M	82	12	1.5	NA	NA	1
14-1396	R	Control	M	82	8	1.5	NA	NA	1
14-1398	R	Control	M	88	9.5	1.5	NA	NA	1
14-0865	R	Control	M	66	10.5	1.66	NA	NA	1

F = female, M = male, POAG = Primary Open Angle Glaucoma, NA = not available.

### Two-photon imaging and cell counting

**Figure 1:**
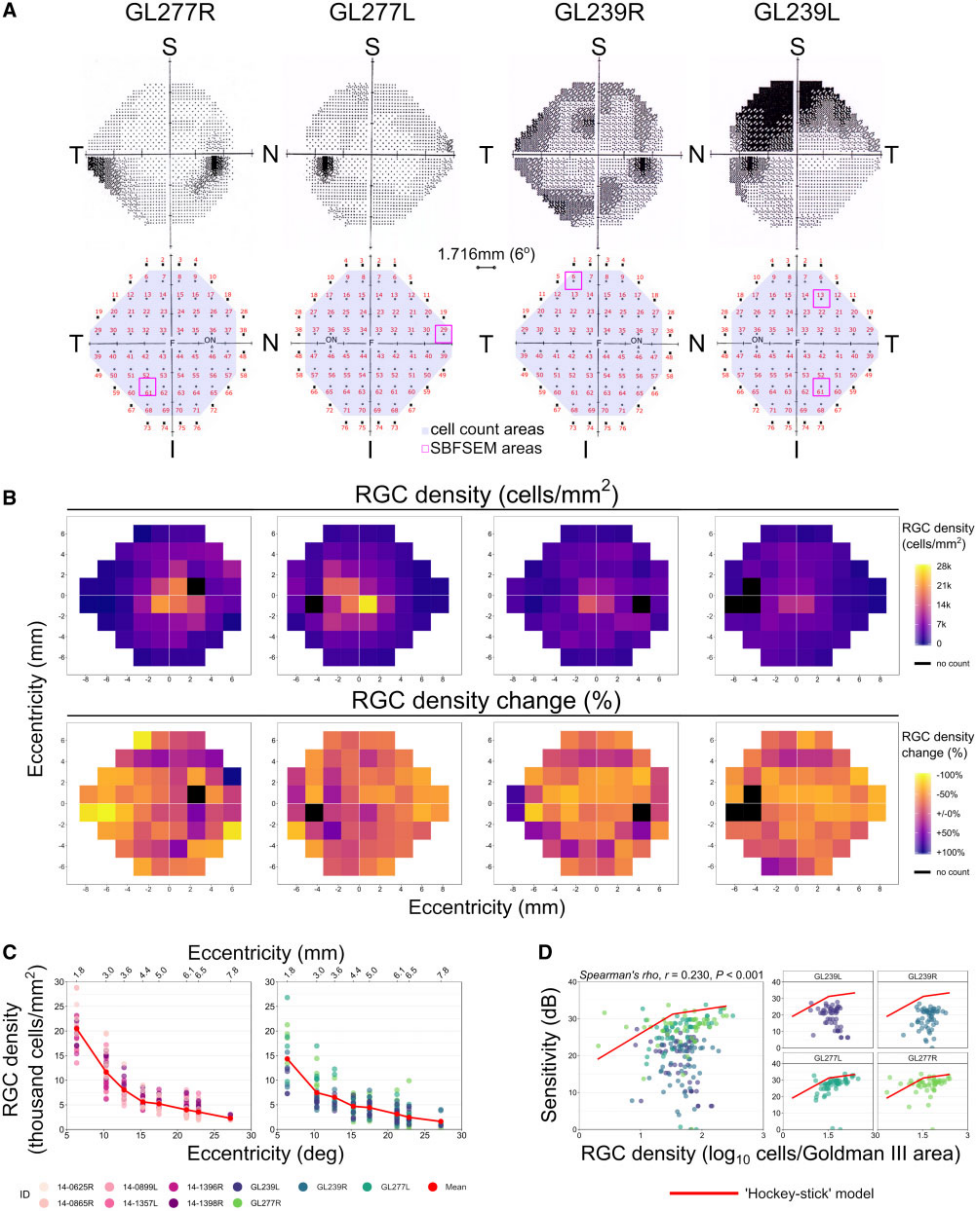
**Retinal ganglion cell loss assessed in relation to visual field deficits.** Human donor eyes from glaucoma donors (*n* = 4) complete with visual field tests conducted prior to death (**A**; *upper row*) were analysed in comparison to control donor eyes (*n* = 6). Whole retinas were imaged by two-photon microscopy, and cell counts made in regions of the retina corresponding to visual field test locations (**A**; *lower row*). Regions were numbered 1–72 beginning superior-temporally, with regions corresponding to visual field test locations highlighted (*purple shading*; *n* = 54 locations). Following imaging, 11 regions were dissected out and processed for SBFSEM (*magenta boxes*). (**B**) Retinal ganglion cell densities were estimated for each test location from *z*-stack counts within an *en face* area of 350 µm^2^ and expressed as cells/mm^2^. Density plots for each glaucomatous retina (**B**; *upper row*) with corresponding percentage change from average control density at each region (**B**; *lower row*). Region locations are inverted along the superior to inferior axis to correspond to the visual field plots (as the superior retina views the inferior visual field and vice versa). Retinal ganglion cell density change against eccentricity was plotted for control (**C**; *left panel*) and glaucoma eyes (**C**; *right panel*). The relationship between retinal ganglion cell density [expressed as log_10_ cells/area of the stimulus (Goldman III)] and visual sensitivity is plotted in **D**. There was no correlation when retinas were grouped as shown by linear regression (‘Spearman’s rho’, *r* = 0.23, *P* < 0.001; **D**). Individual retinas show high variation among eyes (**D**). The ‘hockey stick’ model fitted by Swanson *et al.* (2004) to a plot of normal visual field sensitivity, corrected to a 34-year old (Heijl *et al.*, 1987) against normal retinal ganglion cell counts (Curcio and Allen, 1990), is superimposed on the data from the current study in **D** (*red line*). Individual retinas show a similar relationship when retinal ganglion cell density is high but a greater than expected drop-off in sensitivity when cell density is low. Retinal ganglion cell abbreviated to RGC in **B**–**D**. For **A**, F = fovea, ON = optic nerve; retinal orientation identified by N = nasal, I = inferior, S = superior, T = temporal. Retinal ganglion cell density scales through low (*purple*) to high (*yellow*) (**B**; *upper row*). Retinal ganglion cell density change scales through +100% of control average (*purple*) to −100% of control average (*yellow*) (**B**; *lower row*). Black regions in **B** represent regions around the optic nerve where cell counts were not taken.

Whole retinae (*n *=* *4 for glaucoma, *n *=* *6 for control) were dissected free from the globes and stained overnight at 4°C with 1 µg/ml Hoechst 33342 stain (H1399; Life Technologies). A custom two-photon microscope comprising a moveable objective microscope (MOM^®^; Sutter Instruments) and Ti:Sapphire laser (Mai Tai DeepSee; Newport Spectra-Physics) was used to collect images of 54 retinal regions corresponding to the visual field test locations (Fig. 1A). Retinal distances were taken from the centre of the optic nerve head and measured using a stepper driven stage. Z-stack images (5 µm slices from retinal nerve fibre layer to top of inner nuclear layer) were collected at 40×, giving an *en face* sample area of 350 µm^2^ for each of the 54 regions. All Hoechst-positive nuclei within three sampling regions of 200 × 200 pixels were counted manually using the cell counter plugin for FIJI (Schindelin *et al.*, 2012) and averaged to provide an estimate of cells/mm^2^ for each of the 54 regions. Only round medium-to-large cell nuclei were counted (to exclude vascular endothelial cells identified by their more prolate nuclei). A correction to account for displaced amacrine cells in the ganglion cell layer based on Curcio and Allen (1990) was applied to each test region in control retina. The average number of amacrine cells at each test location was then calculated and subtracted from the cell count for corresponding regions in glaucomatous retina.

### Comparison of retinal ganglion cell density and visual field sensitivity

Pointwise visual field sensitivity values were plotted against log retinal ganglion cell density (cells/mm^2^). Log retinal ganglion cell density was calculated as the number of cells underlying the Goldmann III stimulus (0.431° diameter), using the method outlined by Garway-Heath *et al.* (2000). The area of a Goldmann III stimulus at the fovea is 0.012 mm^2^ (using the conversion factor *q *=* *0.286 mm/°; Garway-Heath *et al.*, 1998). A correction was applied to account for an altered stimulus area with increasing eccentricity from the fovea (Holden and Fitzke, 1988) calculated with the equation: *q*_p_ = *q*_o_ − 0.000014*U*^2^, where *q*_p_ is the conversion factor for locations outside the fovea, *q*_o_ is the conversion factor at the fovea, and *U* is the eccentricity in degrees. Pointwise visual field data were corrected for an expected deterioration in sensitivity as a function of age (but not of disease) between the time of the visual field assessment and death, using the age slopes provided by Heijl *et al.* (1987). To compare with the ‘hockey stick’ model, sensitivity values from the current study were adjusted upwards by 0.9 dB, to account for the expected difference between values from examinations performed with the SITA-Standard strategy and the full threshold strategy.

### Electron microscopy

Eleven retinal regions corresponding to visual field test locations with no-to-moderate cell loss and visual deficit were dissected free (*n *=* *4 retinae, *n *=* *5 regions for glaucoma; *n *=* *4 retinae, *n *=* *6 regions for control). These regions are overlaid on the visual field plots for glaucomatous eyes in Fig. 1A; control regions were matched locations. The retinal regions were washed in 0.2 M sodium cacodylate buffer for 2 days before further fixation in 2.5% glutaraldehyde/2% Paraformaldehyde (PFA) in sodium cacodylate buffer. The tissue was then infiltrated with 1.5% potassium ferricyanide (Arcos)/1% osmium tetroxide (Agar Scientific, UK) in 0.1 M sodium cacodylate buffer, 1% thiocarbohydrazide (Sigma), 1% osmium tetroxide, 1% uranyl acetate (TAAB, Aldermaston, UK) and Walton’s lead aspartate (Agar Scientific) with intermittent washes with distilled water. The tissue was dehydrated through a series of ethanol concentrations followed by propylene oxide and infiltration with Araldite CY212 resin and dodecenyl succinic anhydride mix (Agar Scientific). The resin was changed 14 times over 3 days before embedding the tissue in moulds and curing at 60°C for 48 h. The resin blocks were then trimmed and glued to cryopins (Leica Microsystems Ltd, Milton Keynes, UK). Glass knives were created using a Leica Electron Microscope (EM) KMR2, and the tissue block planed on a UCE ultramicrotome (Reichert-Jung, Cambridge, UK). Conducting carbon cement (Agar Scientific) was applied to avoid charging and resultant damage to the block during imaging before gold coating using an ACE600 sputtercoater (Leica Microsystems). Serial block face scanning electron microscopy (SBFSEM) was performed using a Zeiss Sigma FEG scanning electron microscope (Carl Zeiss, Cambridge, UK), with attached 3View^®^2 system (Gatan, Pleasanton, CA, USA). A total of 500–1000 slices (100 nm thickness) were removed from the block surface by an in-chamber ultramicrotome, each alternating with automated imaging of the remaining block face (79 × 79 µm) to generate an aligned data set with a voxel resolution of 19.2 × 19.2 × 100 nm.

### 3D data analysis

**Figure 2:**
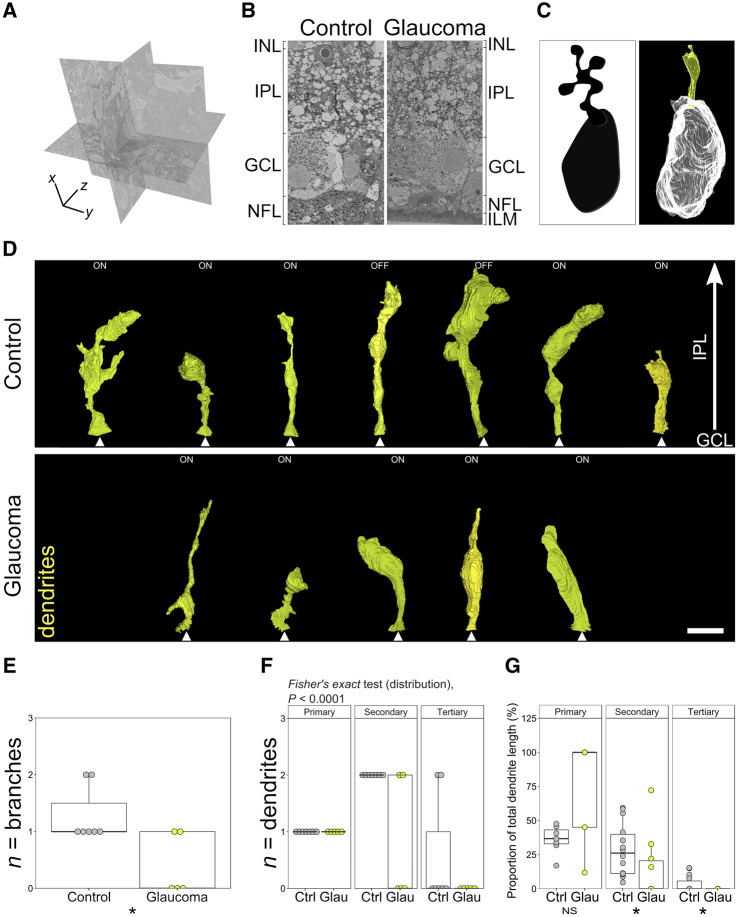
**Dendritic loss is a feature of human glaucoma.** Retinal samples [*n* = 5 from glaucoma (four eyes) and *n* = 6 from controls (four eyes) corresponding to visual field test locations were dissected and prepared for SBFSEM]. Volumetric EM data were generated (**A**) from 79 × 79 × 100 µm tissue cube (19.2 × 19.2 × 100 nm resolution). A representative single slice (*x*–*y* plane) is shown in **B** (cropped in *x*), showing the retinal layers analysed. Retinal ganglion cells were identified, and their dendrites were reconstructed within the IPL. A cartoon of a Golgi-stained midget cell is shown for comparison in **C** alongside a reconstruction from the current SBFSEM data (for more examples see Kolb and Dekorver, 1991). Seven control (**D**, *upper panel*) and five glaucomatous (**D**, *lower panel*) retinal ganglion cells that met inclusion criteria were reconstructed. Dendrites (*yellow*) and synapses (*cyan*) are shown, with the dendrite origin at the soma indicated (*white arrowheads*). Analysis of dendrites demonstrated reduced dendritic branching (**E**) and fewer and shorter secondary and tertiary dendrites in glaucoma, indicating the presence of dendritic atrophy (**F** and **G**; primary dendrites *n* = 5 in glaucoma, *n* = 7 in controls; secondary dendrites *n* = 4 in glaucoma, *n* = 14 in control; tertiary dendrites *n* = 0 in glaucoma, *n* = 3 in control). *P < 0.05, NS = non-significant (*P* > 0.05). For **B**, ILM = inner limiting membrane, NFL = nerve fibre layer, GCL = ganglion cell layer, IPL = inner plexiform layer, INL = inner nuclear layer. Scale bar = 5 μm for **D.**

Retinal ganglion cells were identified and segmented using the TrakEM2 plugin (Cardona *et al.*, 2012) for FIJI. Due to the block size and the relative low cell density, analysis was focused on midget retinal ganglion cells (Fig. 2C, see also; Kolb and Dekorver, 1991; Kolb *et al.*, 1992), the predominant retinal ganglion cell sub-type in the human retina (∼80% of retinal ganglion cells; Dacey, 1993). Dendrites were segmented separately, and the dendritic length was measured. Mitochondria (identified by the presence of cristae) and vacuoles (identified as a double membranous structure without multi-lamellar structures and without easily identifiable electron dense material) were segmented. The Cartesian co-ordinates of each mitochondrion and vacuole were recorded. The distribution of these organelles was measured as the distance from the soma centre along the dendrite and expressed as a Sholl profile (frequency at binned distances), from which an area under the curve was derived. Nearest neighbour distances (NND; as Euclidean distances) were calculated for individual mitochondria and vacuoles within primary, secondary and tertiary dendrites using SPSS NND analysis (*k *=* *3). Individual morphometric analysis of mitochondria was performed using Imaris (Bitplane). New surfaces were generated for individual mitochondria, and volume, sphericity, oblate ellipticity, and prolate ellipticity were calculated for each mitochondrion.

### Statistical analysis

The sample size (*n*) is shown in each figure legend. Graphing and statistical analysis were performed in R and IBM SPSS statistics 23. Statistical tests are indicated at the point of usage within the methods and results. Sholl distributions of mitochondria and vesicles and dendrite composition (primary, secondary and tertiary expressed as a percentage of total) were compared between control and glaucoma using Fisher’s exact test in R. Multiple regression analysis was performed to determine if tissue sampling and quality (eccentricity from macula, time from death to fixation, time in fixation prior to EM processing and donor age; independent variables) influenced dendritic size and branch, mitochondrial and vacuole numbers, volume and distribution (dependent variables). Analysis was performed in R using the lm function (R stats package), and zero-order correlations were calculated using the calc.relimp function (relaimpo package; Grömping, 2006) on *z* scores. For box plots, the centre hinge represents the mean with upper and lower hinges representing the first and third quartiles; whiskers represent 1.5 times the interquartile range. Unless otherwise stated in the figure legends (*P < 0.05, **P <* *0.01, ***P <* *0.001).

### Data availability

All the data are presented in full in this article.

## Results

### Visual sensitivity measured by Standard Automated Perimetry does not accurately predict the degree of retinal ganglion cell loss in glaucoma

Retinal ganglion cell loss in human glaucoma samples was first assessed by two-photon imaging of Hoechst-labelled nuclei. The relationship between cell density and visual sensitivity (identified by Standard Automated Perimetry prior to death) was determined. Two-photon *z*-stack images from whole-mount retinas were collected from 54 regions that corresponded to visual field test locations (Fig. 1A). Average cell counts revealed that cell density in the retinal ganglion cell layer was reduced in glaucomatous eyes by 28% compared with controls when averaged across all regions (*P *<* *0.001; ‘Mann–Whitney’). Heat maps of individual eyes demonstrated that the loss was diffuse and did not qualitatively resemble the corresponding visual field plots (Fig. 1B). Density reduction was best matched in regions where field loss was moderate, but this did not reflect regions of more advanced, arcuate field loss (Fig. 1B). Importantly, the retinal ganglion cell density in the control retinas matched those reported by Curcio and Allen (1990) (Fig. 1C). The relationship between visual field sensitivity and the number of retinal ganglion cells underlying the perimetric stimulus (log_10_ cells per Goldmann III stimulus area) was highly non-linear in eyes with glaucoma (‘Spearman’s rho’, *r *=* *0.23, *P *<* *0.001; Fig. 1C and D). The relationship was similar to the ‘hockey stick’ model in regions where visual deficit was less pronounced (i.e. a shallow relationship when retinal ganglion cell density is high), but a steeper than expected drop-off in sensitivity in regions with lower retinal ganglion cell density (Fig. 1D).

### Retinal ganglion cell dendritic remodelling occurs early in human glaucoma

For detailed analysis of retinal ganglion cell dendrites and their ultrastructure, five regions corresponding to visual field test locations (and six from region- and age-matched control eyes; locations shown in Fig. 1A, *lower row*) were processed for SBFSEM to generate 79 × 79 × 100 µm cubes of retinal data [Fig. 2A; representative single slice (cropped area) shown in Fig. 2B]. Retinal ganglion cells were identified by the presence of an initial axon segment and were required to have a primary dendrite to be processed for further analysis (amacrine cells, the other major cell population of the inner retina, lack an axon, and so were not included). Only midget retinal ganglion cells, comprising ∼80% of the retinal ganglion cell population in the human retina (Dacey, 1993), were processed. In addition, due to their larger dendritic field size, complete parasol retinal ganglion cells were less likely to be captured within individual SBFSEM datasets. Confirmed midget retinal ganglion cells were reconstructed in 3D (example in Fig. 2C) allowing for measurements of dendritic length, volume and surface area. Seven control retinal ganglion cells (five ON centre and two OFF centre; dendrites shown in Fig. 2D, upper panel) and five glaucomatous retinal ganglion cells (five ON centre; dendrites shown in Fig. 2D, *lower panel*) were identified. To account for dendritic field size differences between ON and OFF centre cells, measurements (dendrite, synapse, mitochondria and vacuole) were normalized to the total dendritic length of the cell. There was no significant difference in the average volume of the dendritic tree (*P *=* *0.876) or the average surface area (*P *=* *0.530) in glaucomatous retinal ganglion cells compared with controls; however, retinal ganglion cells from glaucomatous regions of the retina had 69% fewer dendritic branches than those from control tissue (*P *=* *0.048; ‘Mann–Whitney’; Fig. 2E). The proportions of primary, secondary and tertiary dendrites were significantly altered in glaucoma (*P *<* *0.0001; ‘Fisher’s exact’ test; Fig. 2F). Secondary and tertiary dendritic length was reduced suggesting dendrite retraction prior to gross dendritic loss (*P *=* *0.036 and 0.049, respectively; Fig. 2G). To account for changes to dendrites driven by age-related or tissue processing factors, we performed multiple regression analysis. Multiple regression analysis revealed that differences in dendritic branching and length were not driven by variation in tissue fixation times, age of the donor, or the foveal eccentricity of the retinal ganglion cell analysed (**Table 2**). Time to fixation and donor age had a statistically significant effect on variance in dendrite volume [accounting for up to 35% (*P *=* *0.008) and 12% (*P *=* *0.022) of variance, respectively]. However, we saw no statistically significant difference in dendrite volume between control and glaucoma retinal ganglion cells in our morphological analysis.

**Table 2:** Multiple regression analysis

	Dependent variable								
	Dendrite branches	Dendrite volume	Dendrite length	Mito. number	Total mito. volume	Total mito. SAUC	Vacuole number	Total vacuole volume	Total vacuole SAUC
Overall regression model									
*R*^2^	0.685	0.745	0.639	0.508	0.499	0.492	0.489	0.624	0.461
Adj. *R*^2^	0.598	0.599	0.433	0.227	0.212	0.201	0.198	0.409	0.153
*P*	0.060	0.030	0.092	0.232	0.245	0.255	0.258	0.104	0.301
Intercept coef.	1.98	1144.42	204.21	58.37	53.47	374.42	604.94	156.48	3291.80
Independent variables									
Eccentricity									
Variable coef.	−0.02	−3.52	−0.77	1.33	0.04	5.93	−0.66	−0.33	−5.87
Contribution (%)	13.4	27.2	17.5	0.1	5.4	0.2	6.3	22.0	3.2
*P*	0.687	0.228	0.275	0.304	0.884	0.368	0.683	0.409	0.510
Time to fixation									
Variable coef.	0.30	46.29	6.98	7.42	2.92	36.92	8.01	5.51	42.52
Contribution (%)	27.7	35.1	25.7	20.0	26.3	19.0	12.7	34.2	10.7
*P*	0.115	0.008	0.055	0.236	0.069	0.249	0.320	0.019	0.332
Time in fixative									
Variable coef.	0.10	27.33	−0.37	−5.99	0.53	−33.26	−5.67	2.19	−43.01
Contribution (%)	5.7	0.5	8.0	18.9	6.9	18.9	0.3	1.3	0.2
*P*	0.679	0.165	0.940	0.476	0.789	0.443	0.599	0.419	0.470
Age									
Variable coef.	−0.03	−16.96	−2.54	−1.15	−0.87	−6.51	−7.17	−2.27	−38.40
Contribution (%)	21.7	11.7	12.6	11.8	11.3	11.0	29.7	4.8	32.0
*P*	0.663	0.022	0.113	0.676	0.206	0.645	0.074	0.029	0.079

Adj. = adjusted, coef. = coefficient, mito. = mitochondria, SAUC = Sholl area under the curve.

### Changes to mitochondria and vacuoles in glaucomatous retinal ganglion cell dendrites

**Figure 3:**
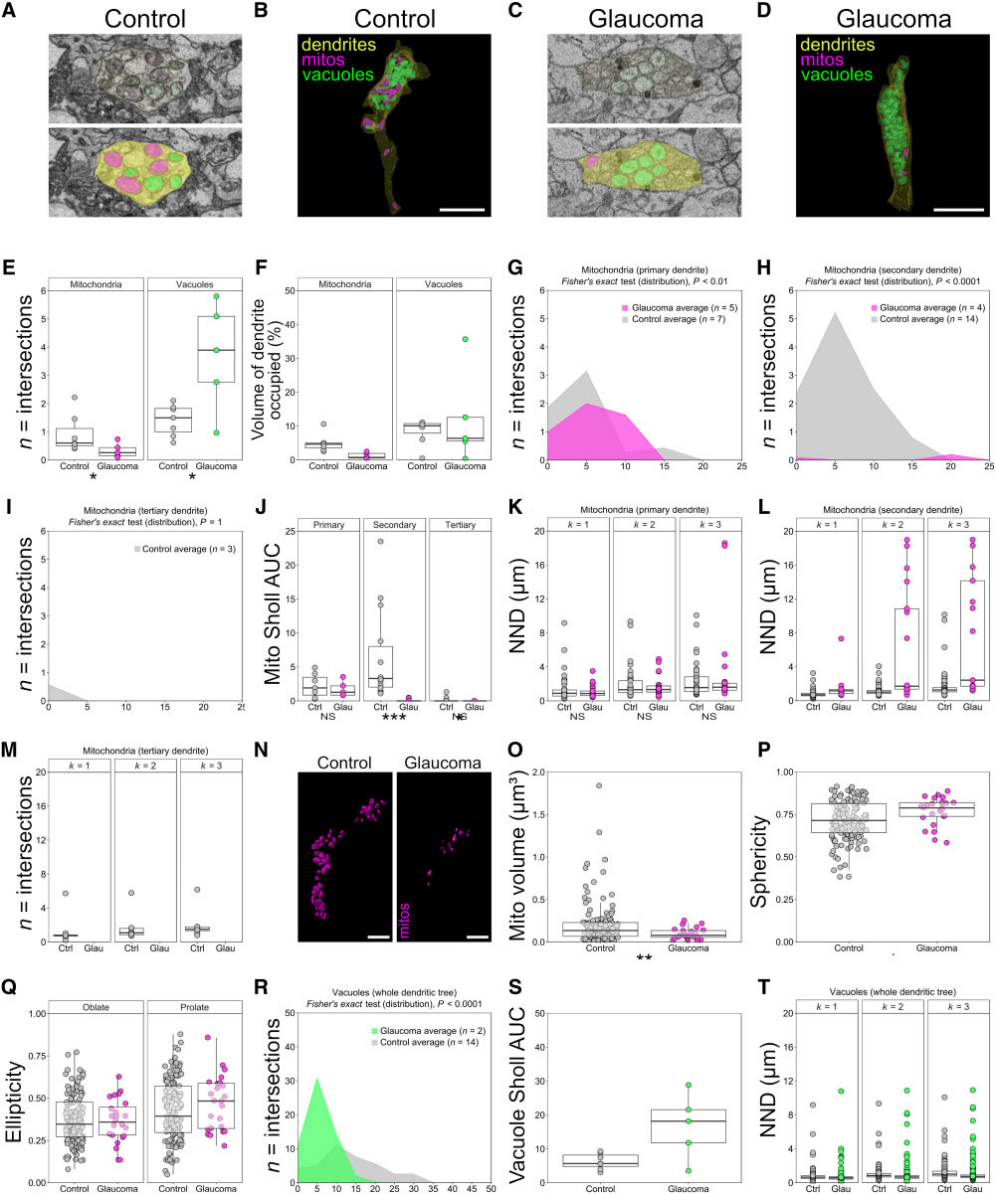
**Mitochondrial morphometry and distribution are altered in human glaucoma.** Organelles from five glaucomatous (*n* = 4 eyes) and seven control retinal ganglion cells (*n* = 4 eyes) were reconstructed in FIJI (ImageJ). Mitochondria (mitos, *magenta*; identified by the presence of cristae; *n* = 198 in controls, *n* = 55 in glaucoma across all cells analysed) and vacuoles (*green*, double membranous structures devoid of cristae and electron dense material; *n* = 273 in controls, *n* = 326 in glaucoma across all cells analysed) were reconstructed within retinal ganglion cell dendrites (*yellow*). Representative EM images for control (**A**) and glaucoma (**C**) and reconstructions (**B** and **D**) are shown. The number of mitochondria per micrometre of dendrite for individual retinal ganglion cells was reduced in glaucoma (**E**), and mitochondria occupied a reduced percentage of dendritic volume in glaucoma compared with controls (**F**). Sholl analysis of mitochondria demonstrates that the mitochondrial distribution across dendrites was altered in glaucoma when expressed as a distribution statistic for primary dendrites (**G**; ‘Fisher’s exact’ test; for individual dendrites, *n* displayed in figure), secondary dendrites (**H**; ‘Fisher’s exact’ test) but not tertiary dendrites (**I**; ‘Fisher’s exact’ test). When expressed as Sholl AUC (**J**), mitochondrial distribution is significantly changed in secondary dendrites in glaucoma compared with controls but not for primary and tertiary dendrites. NND analysis demonstrated no significant change in the proximity of mitochondria to one another in primary dendrites (**K**; mitochondria *n* = 39 in controls, *n* = 38 in glaucoma) but a significant isolation in secondary dendrites [distances to the first, second and third nearest neighbours (*k* = 1–3) increased; **L**; mitochondria *n* = 151 in controls, *n* = 17 in glaucoma]. There were no observable mitochondria in tertiary dendrites in retinal ganglion cells from glaucoma eyes (**M**). Individual mitochondrial surface reconstructions were generated in Imaris (mitochondria *n* = 149 in controls, *n* = 55 in glaucoma), and representative images are shown for control and glaucomatous retinal ganglion cells (**N**). Mitochondria demonstrated significantly reduced volumes in glaucoma (**O**) and were more spherical compared with controls (**P**). Oblate (rounded) and prolate (cigar-shaped) ellipticity was not significantly altered (**Q**). Sholl analysis of vacuoles across the dendritic tree as a whole demonstrated a significant change in distribution (**R**; ‘Fisher’s exact’ test) but not in the Sholl AUC (**S**). The number of vacuoles per micrometre of dendrite was increased in glaucoma (**E**), but the percentage of dendritic volume occupied by vacuoles was unchanged (**F**). Nearest neighbour distances in vacuoles for the *k* = 3 nearest neighbours decreased significantly in glaucoma indicating an increased density of vacuoles (**T**; vacuoles *n* = 273 in controls, *n* = 326 in glaucoma). *P < 0.05, **P < 0.01, ***P < 0.001, NS = non-significant (*P* > 0.05). Scale bar = 5 μm for **B** and **D** and 3 μm for **N.**

Mitochondrial abnormalities and dysfunction are likely to be early features of human glaucoma, and a systemic vulnerability to mitochondrial abnormalities has been reported in glaucoma patients (Osborne *et al.*, 2016*b*). SBFSEM reconstructions allowed the analysis of mitochondrial morphology and distribution within retinal ganglion cell dendrites (examples and reconstructions shown in Fig. 3A–D). Glaucomatous retinas had a reduced number of mitochondria within retinal ganglion cells (−62%, *P *=* *0.048, ‘Mann–Whitney’) and occupied 74% less of the dendritic volume compared with those of controls (*P *=* *0.003; Fig. 3E and F). Mitochondria demonstrated an altered distribution across dendrites (as assessed by Sholl analysis; Fig. 3G–J). Mitochondrial distribution was significantly reduced in secondary dendrites (Sholl area under the curve; *P *<* *0.0001, ‘Mann–Whitney’) with a significantly altered distributions across the Sholl analyses (*P* < 0.0001; ‘Fisher’s exact’ test; Fig. 3H and J). NND analysis of individual mitochondria within dendrites demonstrated no significant increase in distance between the three nearest neighbours (*k *=* *3) for mitochondria within primary dendrites (*k *=* *1, *P *=* *0.711; *k *=* *2, *P *=* *0.901; *k *=* *3, *P *=* *0.921; ‘Mann–Whitney’) but a substantial increase in NND in secondary dendrites (*P *<* *0.0001 for *k *=* *1, *k *=* *2 and *k *=* *3) indicating that mitochondria become increasingly isolated in more peripheral dendritic segments (Fig. 3K–M).

We next assessed the morphology of individual mitochondria within retinal ganglion cells. Smaller mitochondria have previously been observed in ophthalmic and neurodegenerative diseases (including glaucoma) and are typically indicative of a mitochondrial fusion/fission imbalance and/or an imbalance in mitophagy (Knott *et al.*, 2008; Kong *et al.*, 2009; Williams *et al.*, 2012, 2017*b*; Coughlin *et al.*, 2015). Shape factor analysis revealed that individual mitochondria were smaller (53%, *P *=* *0.008, ‘Student’s *t*-test’) and more rounded (9%, *P *=* *0.010) in glaucomatous retinal ganglion cells compared with those in controls (Fig. 3N–P). This is consistent with mitochondrial morphological changes demonstrated in model hypoxia systems (Campello and Scorrano, 2010; Ahmad *et al.*, 2013; Fuhrmann and Brune, 2017; Zhang *et al.*, 2018), and other groups have demonstrated that glaucomatous retinal ganglion cells likely undergo chronic intermittent hypoxia following periods of elevated intraocular pressure (Tezel and Wax, 2004; Holcombe *et al.*, 2008; Chidlow *et al.*, 2017). Mitochondria in glaucomatous retinal ganglion cells had no significant changes to oblate (rounded; 1%, *P *=* *0.984) or prolate ellipticity (cigar shaped; 11%, *P *=* *0.76; Fig. 3Q).

Other double membranous structures (within the size range of 0.1–5 µm) were grouped and segmented. These comprised small vacuoles, granulovacuolar degeneration bodies containing only small quantities of electron dense material and mitochondria devoid of cristae structure (examples in Fig. 3A and C). The appearance of vacuoles is an age- and disease-related change observed in neurons in aged rodents (de Estable-Puig and Estable-Puig, 1975) and post-mortem human tissue from various dementias (Ball and Lo, 1977; Nakamori *et al.*, 2013). Granulovacuolar degeneration bodies, a hallmark of Alzheimer’s disease, are late-stage autophagocytic vacuoles, the accumulation of which may relate to incomplete autophagy (Funk *et al.*, 2011). Mitochondria devoid of cristae have been reported in animal models of glaucoma (Coughlin *et al.*, 2015; Williams *et al.*, 2017*b*) and other neurodegenerations (Baloyannis, 2011; Williams *et al.*, 2012; Franco-Iborra *et al.*, 2018). In the present study, we could not confidently separate these structures and so grouped them as vacuoles to generate a meaningful index of degeneration. We observed that these vacuoles occupied 12% of the dendritic volume in glaucoma and 8% in control retinal ganglion cells (*P *=* *1.00, ‘Mann–Whitney’; Fig. 3E and F). These structures increased in frequency by 40% in glaucomatous retinal ganglion cells compared with controls (*P *=* *0.048, ‘Mann–Whitney’) and were distributed along a higher proportion of the dendritic tree (as shown by Sholl area under the curve; 62% increase, *P *=* *0.073, ‘Mann–Whitney’; and as assessed by ‘Fisher’s exact’ test, *P *<* *0.0001; Fig. 3R and S). NND analysis (Fig. 3T) demonstrated a decrease in distance between individual vacuoles in glaucomatous retinal ganglion cells (*P *<* *0.0001 for *k* = 1, *k* = 2 and *k* = 3; ‘Mann–Whitney’). These findings are consistent with imbalanced fusion/fission and autophagy, and are an indicator of increased neurodegenerative insults in glaucomatous retinal ganglion cells.

We performed multiple regression analysis to determine whether age-related or tissue processing factors influenced mitochondria and vacuole metrics. Multiple regression analysis demonstrated that differences in mitochondrial and vacuole numbers, volume and distribution were not driven by variation in tissue fixation times, age of the donor or the foveal eccentricity of the retinal ganglion cell analysed (**Table 2**). No statistically significant model was produced, and all showed weak correlation (all *R*^2^ < 0.625, *P *>* *0.1). Time to fixation and donor age had a statistically significant effect on variance in total vacuole volume [accounting for up to 34% (*P *=* *0.019) and 5% (*P *=* *0.029) of variance, respectively]. The magnitude of change between control and glaucoma vacuole volume may therefore be overestimated in these data due to the variability of tissue but are not the predominant determinant of difference.

Collectively, these data demonstrate that following periods of elevated intraocular pressure, retinal ganglion cell dendrites degenerate prior to gross cell loss with marked changes to mitochondrial frequency and density.

## Discussion

Retinal connectomics by electron microscopy or genetic labelling has established itself as the technique to dissect neuronal tracts and pathways in detail. These studies are usually undertaken using specially prepared tissue from model animals, in either normal or developing retina (Briggman *et al.*, 2011; Helmstaedter *et al.*, 2013; Kim *et al.*, 2014; Marc *et al.*, 2014; Greene *et al.*, 2016). Due to the time to fixation, human donor tissue from glaucoma patients has typically been of insufficient quality to enable neuronal analysis. A short time to fixation is key in preserving neuronal integrity, but prolonged fixation raises considerable technical challenges with standard techniques for visualizing neurons (e.g. Golgi staining, intracellular filling and DiOlistic labelling; Dowling, 2012; Balendra *et al.*, 2015). We sought to overcome these challenges by using rare tissue with a shorter post-death time to fixation (average ∼6 h) than typical for post-mortem tissue, and specifically selecting regions of retina with known clinical history (in particular, visual field sensitivity data) and known retinal ganglion cell densities (determined histologically).

Retinal ganglion cell degeneration in glaucoma is a compartmentalized process, with different factors affecting different compartments of the cell (axon, soma, dendrites, synapses, and mitochondria; Whitmore *et al.*, 2005; Della Santina and Ou, 2017; Williams *et al.*, 2017*a*). However, our knowledge of this compartmentalized degeneration comes primarily from animal models with only limited data from human tissue (Wang *et al.*, 2003; Morrison *et al.*, 2005). In our dataset, human glaucomatous retinal ganglion cells undergo dendritic and mitochondrial changes. Given the presence of dendritic degeneration, it is reasonable to conclude that these RGCs have less synaptic contacts, but this could not be definitively assessed in these datasets due to the available resolution of SBFSEM when capturing large image volumes. Further studies on single retinal ganglion cells with higher resolution and higher throughput methods are warranted to explore synapses at this level. Synapse loss prior to marked neurodegeneration has been demonstrated in animal models of glaucoma (Della Santina *et al.*, 2013; Berry *et al.*, 2015; Williams *et al.*, 2016) and is a common feature of other neurodegenerations (Reddy *et al.*, 2012; Williams *et al.*, 2012). The process is predicted to be an early driver of visual dysfunction in glaucoma.

Reduction in mitochondrial volume and distribution may indicate that glaucomatous retinal ganglion cells are under increased metabolic strain, an emerging aspect of early glaucomatous degeneration in animal models (Baltan *et al.*, 2010; Ebneter *et al.*, 2011; Osborne *et al.*, 2016*b*; Inman and Harun-Or-Rashid, 2017; Williams *et al.*, 2017*a*, *b*). Furthermore, genomic analysis has demonstrated increased mitochondrial DNA content and a spectrum of mitochondrial DNA mutations in glaucoma patients (Chrysostomou *et al.*, 2013; Van Bergen *et al.*, 2015; Zhang *et al.*, 2016). In addition, many diseases caused by mutations in mitochondrial genes, or nuclear genes encoding mitochondrial proteins, primarily affect retinal ganglion cells and present as visual disorders with little or no extra-ophthalmic symptoms (e.g. *MT-ND1*, *MT-ND4* and *MT-ND6* in Leber’s hereditary optic neuropathy; *OPA1* in autosomal dominant optic atrophy; Yu-Wai-Man *et al.*, 2011; Pilz *et al.*, 2017), suggesting retinal ganglion cell sensitivity to mitochondrial perturbations (Williams *et al.*, 2012).

The majority of cells in our SBFSEM datasets were devoid of dendrites, which likely arose from the delay between death and fixation, where further neuronal degeneration may have occurred. We have previously demonstrated that axotomized rodent retina maintained in culture following extraction exhibit dendritic atrophy within 6 h (Binley *et al.*, 2016). However, fresh, axotomized human retina explant tissue culture has proven to be a useful tool to assess neurodegenerative insults and treatments despite this initial wave of degenerative changes (Osborne *et al.*, 2016*a*, 2018). The mean time from death to fixation was shorter in the glaucoma eyes compared with controls (3.1 ± 1.1 and 7.33 ± 1.2 h), while control eyes were from older donors than glaucoma eyes. It is therefore reasonable to consider that dendritic degeneration may have been underestimated in the glaucoma eyes. Multiple regression analysis demonstrated that time from death to fixation may have contributed to changes in dendritic volume, but the differences observed between control and glaucomatous retinal ganglion cells were predominantly related to factors other than tissue preparation. Intact, albeit, degenerated dendrites are an indication that potentially functional cells remain in regions of the retina that have no-to-moderate cell loss and visual deficits.

We observed a reduction in the number of mitochondria, with changes in size, shape and distribution across the dendritic tree. We also observed an increase in double membranous structures, which likely include vacuoles, granulovacuolar degeneration bodies and mitochondria devoid of cristae structure. While these structures are characteristics of neurodegenerative processes, we cannot exclude the possibility that they occur as part of the normal aging process (as these eyes came from significantly aged individuals) or that the rate and magnitude of the appearance of these structures differ in a disease context. Since they were also present in control tissue, it is possible that they are artefacts arising from the delay in death to fixation and the storage of tissue long term in PFA (as opposed to osmium tetroxide or cacodylate buffer as standard in electron microscopy preparations). Multiple regression analysis demonstrated that differences in tissue fixation time had no significant relationship to mitochondria or vacuole variance, but time from death to fixation may have contributed to changes in vacuole volume. This suggests that the difference between control and glaucomatous retinal ganglion cell vacuole volume may be overestimate, but it remains predominantly related to factors other than tissue quality. However, the definitive evaluation and staging of this process would require tissue available at post-mortem stages that are unrealistic. Conservatively, our data support the neurodegenerative processes observed in animal models. Further studies with larger numbers of eyes would be required to provide more detailed quantification of the relationship between these degenerative changes and visual dysfunction.

The compartmentalized degeneration of retinal ganglion cells results in functional visual loss in animal models of glaucoma (Weber and Harman, 2005; Howell *et al.*, 2007; Della Santina *et al.*, 2013). These data suggest that in humans, early degenerative changes prior to gross axon and soma loss contributes to vision loss. The ‘hockey stick’ model of Swanson *et al.* (2004) predicts that the rate of change in sensitivity with respect to retinal ganglion cell number is low when the stimulus is larger than the critical summation area but becomes greater when the stimulus is smaller than the critical summation area. This model applies in healthy observers as the critical summation area enlarges with increasing eccentricity, encompassing a constant number of retinal ganglion cells. Although it is known that the critical summation area is enlarged at fixed locations in the visual field in glaucoma (Redmond *et al.*, 2010), the range over which it can enlarge is unknown. The steeper than expected drop-off in sensitivity at lower retinal ganglion cell densities observed in the present study (Fig. 1D) would suggest that the range of enlargement is shorter in glaucoma than that observed across eccentricities in healthy controls. A number of difficulties with the limitations of Standard Automated Perimetry and cell counting may have contributed to the discrepancy found in the current study. Inter- and intra-test variabilities substantially increase with the depth of visual field defect (Henson *et al.*, 2000; Artes *et al.*, 2002), which may increase variance in the functional data. Fixational drift and microsaccades can vary the location of individual stimuli (Henson *et al.*, 1996), and if retinal ganglion cell loss is heterogeneous, this could manifest as greater variability in sensitivity measurements. Pre-neuronal abberations (e.g. optical defects) could also influence sensitivity. Nuclear counts will not distinguish between functional retinal ganglion cells and those with reduced or absent visual input (i.e. synapse and axon loss, which we addressed further in our SBFSEM studies presented here). The interval from the last visual field to time of death was 7–21 months, in which time retinal ganglion cell degeneration could have progressed, but given that regions of no significant cell loss demonstrated a visual deficit, this seems an unlikely source of discrepancy. The degree of cell loss within visual field test areas has previously been shown to have a weak relationship with visual sensitivity (*R*^2^ = 0.31; Kerrigan-Baumrind *et al.*, 2000). In these experiments, cell counts of four retinal sections from 28 test locations were conducted across 17 eyes; however, these data are caveated as the linear regression fits were calculated against sensitivity (dB scale; a log scale) without log scaling of retinal ganglion cell number and performed on retinal sections rather than whole-mounted retina. The present study suggests that visual field sensitivity is lower, overall, than expected from the ‘hockey stick’ model for a range of retinal ganglion cell densities, with considerable variance in the sensitivity data, particularly in locations with lower retinal ganglion cell density. Taken together, the findings of the current study suggest that in glaucoma, deficits at the level of the single cell may initially be masked when retinal ganglion cell density is high and contribute to the steeper than expected drop-off in sensitivity when the density is low.

The detection of the earliest signs of retinal damage remains a sought after target for clinicians managing glaucoma. The detection of retinal ganglion cell apoptotic events using fluorescent annexin-V tagging suggests one possibility (Cordeiro, 2007; Cordeiro *et al.*, 2017). However, the cells identified by this method are already undergoing cell death, the temporal dynamics of which are still unresolved, and, therefore, are unlikely to be targets for functional recovery. The demonstration of changes in dendritic and mitochondrial structures and distribution in the present study suggest that these may present a clinically detectable target of early retinal neuronal damage. While these structures fall within the theoretical detection limit for high-resolution OCT (Morgan *et al.*, 2017), direct imaging is unlikely in view of their relatively low contrast. However, changes in optical scatter induced by neurodegenerative changes in the inner retina are a realistic target. We have demonstrated that changes in optical texture of the inner plexiform layer can be detected by high-resolution OCT and correlated with histological changes in retinal explants (Tudor *et al.*, 2014). Preliminary work in human glaucoma suggests that optical texture, as derived by OCT, can support the early diagnosis of glaucoma (Anantrasirichai *et al.*, 2013). In addition, there is increasing evidence for the detection of Alzheimer’s disease by OCT of the inner retina (den Haan *et al.*, 2017; Liao *et al.*, 2018). Mitochondrial loss has been reported in retinal ganglion cells in mice carrying Alzheimer’s disease-associated mutations (Williams *et al.*, 2013*b*) and may therefore also provide optical texture changes for earlier and more sensitive detection of Alzheimer’s disease.

In conclusion, we demonstrate a proof-of-concept, first-in-glaucoma, high-resolution analysis of glaucomatous retinal ganglion cells permitting the analysis of dendritic and mitochondrial structures. Further studies with an increased number of cells across various glaucoma pathologies will allow the delineation of the extent to which these changes are common across glaucoma subgroups.

## Abbreviations

NND: nearest neighbour distance
OCT: optical coherence tomography
SBFSEM: serial block face scanning electron microscopy
